# Supplementary material for: Health Professionals’ Perceptions of Pacific Co-Designed Resources for Pacific Gout Patients
Source: Healthcare (Basel). 2025 Aug 22;13(17):2089. doi: 10.3390/healthcare13172089 (PMC12427854; doi:10.3390/healthcare13172089)
Supplement: Supplementary file 1 [file healthcare-13-02089-s001.zip › healthcare-3775158-supplementary.pdf]

Supplementary file S1: Table of Consolidated criteria for reporting qualitative research checklist

No.	Item	Guide questions/ description	Checklist of this study
**Domain 1: Research team and reflexivity**			
**Personal Characteristics**			
1.	Interviewer/facilitator	Which author/s conducted the interview or focus group?	Three authors (M.T, S.O, M.O.).
2.	Credentials	What were the researcher's credentials? E.g. PhD, MD	S. Ofanoa (PhD)<br>M. Ofanoa (PhD)<br>M. Tohi (PhD)
3.	Occupation	What was their occupation at the time of the study?	Lecturer, research fellow, and research assistant.
4.	Gender	Was the researcher male or female?	S. Ofanoa (male)<br>M. Ofanoa (male)<br>M. Tohi (female)
5.	Experience and training	What experience or training did the researcher have?	The interviewers have all had extensive training in qualitative research methods.
**Relationship with participants**			
6.	Relationship established	Was a relationship established prior to study commencement?	No
7.	Participant knowledge of the interviewer	What did the participants know about the researcher? e.g., personal goals, reasons for doing the research	Reasons for doing the research.
8.	Interviewer characteristics	What characteristics were reported about the interviewer/facilitator? e.g. Bias, assumptions, reasons and interests in the research topic	Reasons for doing the research and interests in the research topic.
**Domain 2: study design**			
**Theoretical framework**			

9.	Methodological orientation and Theory	What methodological orientation was stated to underpin the study? e.g., grounded theory, discourse analysis, ethnography, phenomenology, content analysis	Reflexive thematic analysis
**Participant selection**			
10.	Sampling	How were participants selected? e.g., purposive, convenience, consecutive, snowball	Purposive sampling technique
11.	Method of approach	How were participants approached? e.g., face-to-face, telephone, mail, email	Online Zoom
12.	Sample size	How many participants were in the study?	14 participants
13.	Non-participation	How many people refused to participate or dropped out? Reasons?	None
**Setting**			
14.	Setting of data collection	Where was the data collected? e.g., home, clinic, workplace	Online via Zoom
15.	Presence of non-participants	Was anyone else present besides the participants and researchers?	No one else was present apart from the participants and researchers.
16.	Description of sample	What are the important characteristics of the sample? e.g., demographic data, date	Demographic data
**Data collection**			
17.	Interview guide	Were questions, prompts, guides provided by the authors? Was it pilot tested?	A guide was prepared and it was piloted.
18.	Repeat interviews	Were repeat interviews carried out? If yes, how many?	No

19.	Audio/visual recording	Did the research use audio or visual recording to collect the data?	Research used audio recording.
20.	Field notes	Were field notes made during and/or after the interview or focus group?	Field notes were made during the interview and used to support analysis.
21.	Duration	What was the duration of the interviews or focus group?	Ranged between 40 to 60 minutes.
22.	Data saturation	Was data saturation discussed?	Yes, further discussed in the Methods Section.
23.	Transcripts returned	Were transcripts returned to participants for comment and/or correction?	No
**Domain 3: analysis and findings**			
**Data analysis**			
24.	Number of data coders	How many data coders coded the data?	Two (S.O & S.T)
25.	Description of the coding tree	Did authors provide a description of the coding tree?	Yes
26.	Derivation of themes	Were themes identified in advance or derived from the data?	Derived from the data.
27.	Software	What software, if applicable, was used to manage the data?	NVivo software
28.	Participant checking	Did participants provide feedback on the findings?	No
**Reporting**			
29.	Quotations presented	Were participant quotations presented to illustrate the themes / findings? Was each quotation identified? e.g., participant number	Yes

30.	Data and findings consistent	Was there consistency between the data presented and the findings?	Yes
31.	Clarity of major themes	Were major themes clearly presented in the findings?	Yes
32.	Clarity of minor themes	Is there a description of diverse cases or discussion of minor themes?	Yes

<u>Supplementary file S2: Semi-structured talanga /interview schedule</u>

Key prompts and questions

Practitioner's current views & practice

- Views on current awareness/understanding of gout for Pacific patients
- Barriers to understanding or awareness that you see in your practice
- Your experiences when providing health education about gout to patients
- Existing resources available in the practice for gout patients

Introduce the community designed resources: video & brochures

What are your thoughts/views on the co-designed resources?

- Views on the video format vs the brochure format
- Acceptability of the resources for practitioners & for patients
- Views on its feasibility for the clinical setting
    - Ways of using the resources to provide health education about gout
- Potential strengths and barriers of using this resource in your work
- Overall feedback on the resources
- Other comments/or things they wish to add

Wrap up

Final comments
